# Airway autoimmune responses in severe eosinophilic asthma following low-dose Mepolizumab therapy

**DOI:** 10.1186/s13223-016-0174-5

**Published:** 2017-01-06

**Authors:** Manali Mukherjee, Hui Fang Lim, Sruthi Thomas, Douglas Miller, Melanie Kjarsgaard, Bruce Tan, Roma Sehmi, Nader Khalidi, Parameswaran Nair

**Affiliations:** 1 Department of Medicine, McMaster University & St. Joseph’s Healthcare, Hamilton, ON Canada; 2 Department of Respiratory Medicine, National University of Singapore, Singapore, Singapore; 3 Department of Otolaryngology, Northwestern University, Feinberg School of Medicine, Chicago, IL USA; 4 Firestone Institute for Respiratory Health, 50 Charlton Avenue East, Hamilton, ON L8N 4A6 Canada

**Keywords:** Mepolizumab, Autoantibodies, Autoimmune, Eosinophilic asthma, IL-5, Sputum, Immune complex

## Abstract

**Background:**

Anti-interleukin (IL)-5 monoclonal antibodies as an eosinophil-depleting strategy is well established, with Mepolizumab being the first biologic approved as an adjunct treatment for severe eosinophilic asthma.

**Case presentation:**

A 62-year old woman diagnosed with severe eosinophilic asthma showed poor response to Mepolizumab therapy (100 mg subcutaneous dose/monthly) and subsequent worsening of symptoms. The treatment response to Mepolizumab was monitored using both blood and sputum eosinophil counts. The latter was superior in assessing deterioration in symptoms, suggesting that normal blood eosinophil count may not always indicate amelioration or adequate control of the ongoing eosinophil-driven disease process. This perplexing situation of persistent airway eosinophilia and increased steroid insensitivity despite an anti-eosinophil therapy can be explained if the administered dose of the mAb was inadequate in comparison to the target antigen. The resultant immune complexes could act as ‘cytokine depots’, protecting the potency of the ‘bound’ IL-5, thereby sustaining the eosinophilic inflammation within the target tissue. Molecular analysis of the sputum indicated the development of a polyclonal autoimmune response as well as an increase in group 2 innate lymphoid cells, two novel observations in severe eosinophilic asthma, which were associated with indices of disease severity and progression. This case highlights the possibility of a previously unrecognised autoimmune-mediated worsening of asthma perhaps triggered by immune complexes formed due to inadequate dosing of administered monoclonal antibodies in the target tissue.

**Conclusions:**

While anti-IL5 mAb therapy is an exciting novel option to treat patients with severe asthma, there is the rare possibility of worsening of asthma as observed in this case study, due to local autoimmune mechanisms precipitated by potential inadequate airway levels of the monoclonal antibody.

**Electronic supplementary material:**

The online version of this article (doi:10.1186/s13223-016-0174-5) contains supplementary material, which is available to authorized users.

## Background

The past decade has witnessed the development of several anti-cytokine monoclonal antibody therapies (mAb) for asthma, with Mepolizumab, an IgG_1_ mAb against IL-5, being the first biologic approved for severe eosinophilic asthma [1]. We report a worrying scenario of asthma worsening, following 100 mg subcutaneous (s.c) Mepolizumab therapy in a patient with severe eosinophilic asthma. In this article we draw attention to two factors: (i) enumerating eosinophils in sputum is more useful to monitor treatment response than in blood; (ii) low-dose mAb therapy might lead to increased inflammation triggered by in vivo immune complex (IC) formation between drug and the target cytokine (IL-5), when the latter is in excess to the former in the target tissue. This is more likely to affect patients whose asthma is severe enough to require maintenance systemic corticosteroids to control their airway eosinophilia.

## Case presentation

**Fig. 1:**
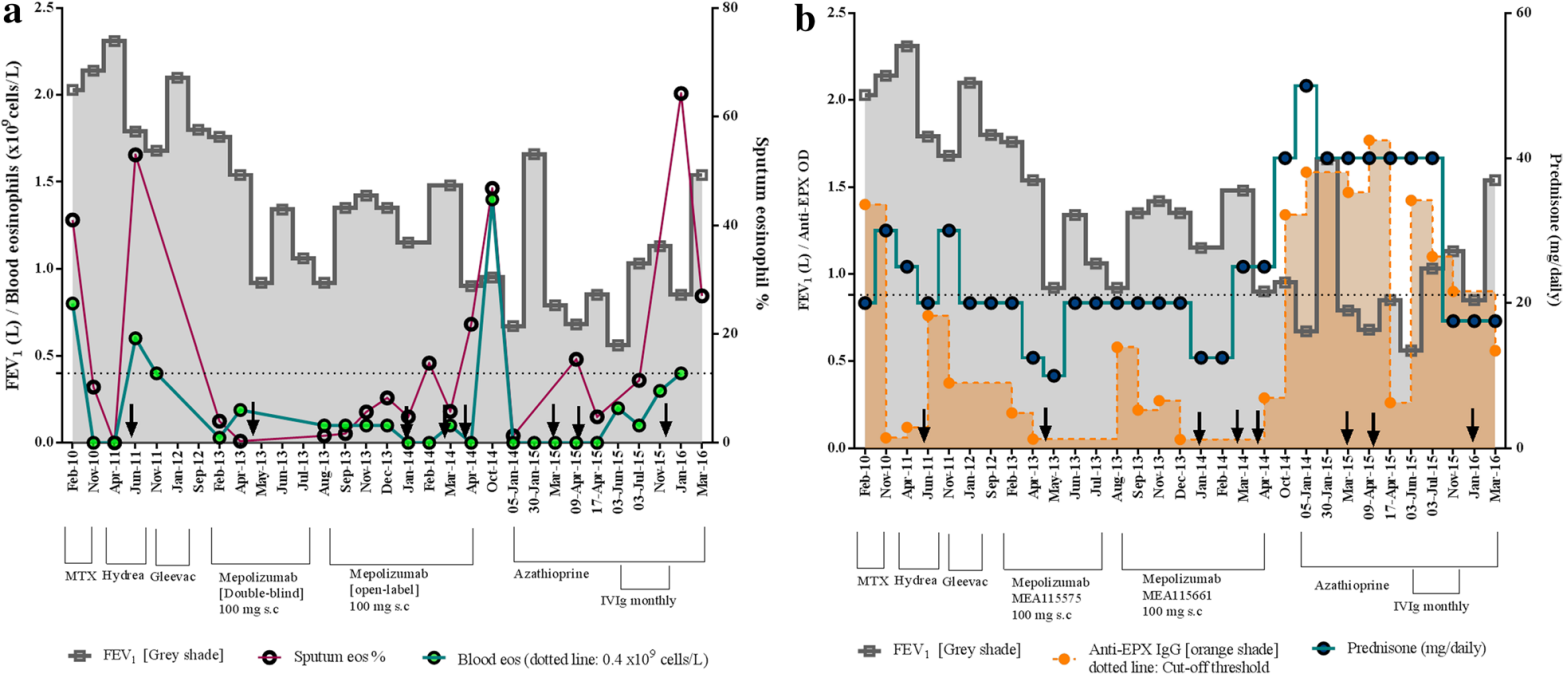
Disease progression timeline of treatments, development of autoimmune response and clinical severity indices. **a** FEV_1_ and blood eosinophils (10^9^/L) is plotted on the *left Y axis*, while sputum eosinophil % on the *right Y axis* for the given time points. *Dotted line* represents the upper-limit of normal blood eosinophil level. **b** Association of FEV_1_ and sputum anti-EPX IgG (see Additional file 1: online repository for methods) is plotted on the *left Y axis*, while daily prednisone dose is plotted on the *right Y axis*. For both, *black arrow* indicates intravenous solumedrol (except last indicated on Jan-16 refers to 40 mg prednisone burst, tapered down to maintenance 17.5 mg dose)

A 62-year old non-atopic woman, with seven pack-year smoking history, and adult-onset asthma (diagnosed at 21 years) whose symptoms worsened at the age of 55 was seen in our clinic on February 22nd, 2010 with severe airway hyper-responsiveness (PC_20_ methacholine <0.03 mg/mL), mild airflow obstruction (FEV_1_ 2.04 L, 75% predicted, FEV_1_/VC 75%), and chronic rhinosinusitis with polyposis. The eosinophilic nature of her asthma was confirmed by peripheral blood counts (peaked at 0.8 × 10^9^/L in 2010) and sputum cellularity (eosinophils >3% of total cell count with free granules on multiple occasions). She did not have mutations for PDGFR-FIP1L1, c-kit, JAK2, or BCR-Abl or abnormal lymphocyte population or T cell receptor rearrangements. Her routine chemistry, total serum IgE, and tryptase were normal, as were her stool microscopy, antifungal precipitins, and autoantibody profile including cytoplasmic and perinuclear anti-neutrophil cytoplasmic antibodies. Computed tomography of thorax was unremarkable. She had two sinus polypectomies that did not improve her respiratory symptoms significantly. She has been prednisone-dependent since 2008. Methotrexate, hydroxyurea, and imatinib were not effective to wean her off prednisone (Fig. 1). The patient was known to be compliant with her medications, and her inhaler technique was deemed adequate.

By 2013, she required a daily dose of 2500 mcg fluticasone propionate, long-acting beta-2 agonists, muscarinic antagonists, and 20 mg prednisone to maintain an FEV_1_ of 1.76 L (65% of predicted), blood eosinophils 0.03 × 10^9^ cells/L, and 4% sputum eosinophils (Fig. 1a). With four exacerbations in the preceding year, she was enrolled into a double-blinded placebo controlled Mepolizumab clinical trial (#MEA115575) (in which she received the active drug), followed by an open-label extension (#MEA115661). In the double-blinded trial, her FEV_1_ was 1.76 L at the start of the study (Feb-13) that dropped to 0.9 L at the end of the study (Aug-13), with no demonstrable steroid-sparing effect (Fig. 1a). In the open-label extension, she received nine monthly infusions of 100 mg s.c Mepolizumab, without an improvement in her FEV_1_, and two interim courses of intravenous solumedrol to manage her deteriorating symptoms. The anti-eosinophil effect of Mepolizumab was apparent from her depleting blood eosinophil levels and her sputum eosinophils being maintained below 3% until September 2013 (Fig. 1a). The initial drop in her FEV_1_ was therefore not eosinophil-driven, indicating the presence of alternative mechanisms. Furthermore, her lung function continued to deteriorate with increasing airway eosinophilia (not reflected in blood), and prednisone requirement that now doubled from a pre-study dose of 20–40 mg daily (Fig. 1b). The patient did not develop any circulating anti-Mepolizumab antibodies that could explain this.

## Discussion and molecular insights

Mepolizumab is an effective therapy to reduce blood and sputum eosinophils in severe prednisone-dependent asthma, with a good safety profile and low incidence of circulating anti-drug antibodies [1–3]. However, the magnitude of clinical efficacy may be lower regarding prednisone-sparing effect with 100 mg of the drug administered subcutaneously compared to 750 mg intravenously. This may be due to inadequate concentrations of the drug in the airway. We are unable to confirm this as the mAb pharmacokinetics in the airway of asthmatics has not been established. It is plausible, in the event of inadequate dosing in the airways of patients with high IL-5 concentration, drug-antigen IC clusters can form (Additional file 1: Figure S1 in the online repository). Being an IgG_1_ humanised mAb, the IL-5/Mepolizumab ICs, can precipitate, bind complement and elicit further inflammation and tissue injury. In the complexed form, a neutralising mAb protects the active-site of the cytokine from in vivo degradation [4]. This increases the bioavailability of IL-5 to its target receptors present on tissue-resident cells like ILC2s and eosinophil progenitors (EoPs). Enumeration of both these cell types was recently reported to be significantly higher in severe asthmatic airways [5].

This case study offered us a unique opportunity to investigate this hypothesis of an IC-mediated worsening of patient symptoms in the event of inadequate mAb dosing. Due to unavailability of a biopsy sample, tissue deposition of ICs could not be performed. An attempt to quantify complement consumption in the sputum to assess local IC formations was inconclusive. The assay performance with sputum supernatants was unreliable in comparison to other biological samples like nasal polyp extracts performed alongside. Nevertheless, a series of molecular investigations were undertaken to answer this perplexing situation of persistent airway eosinophilia (but normal blood eosinophil) while on treatment with Mepolizumab, deteriorating lung function and subsequent increase in steroid-insensitivity.

**Fig. 2:**
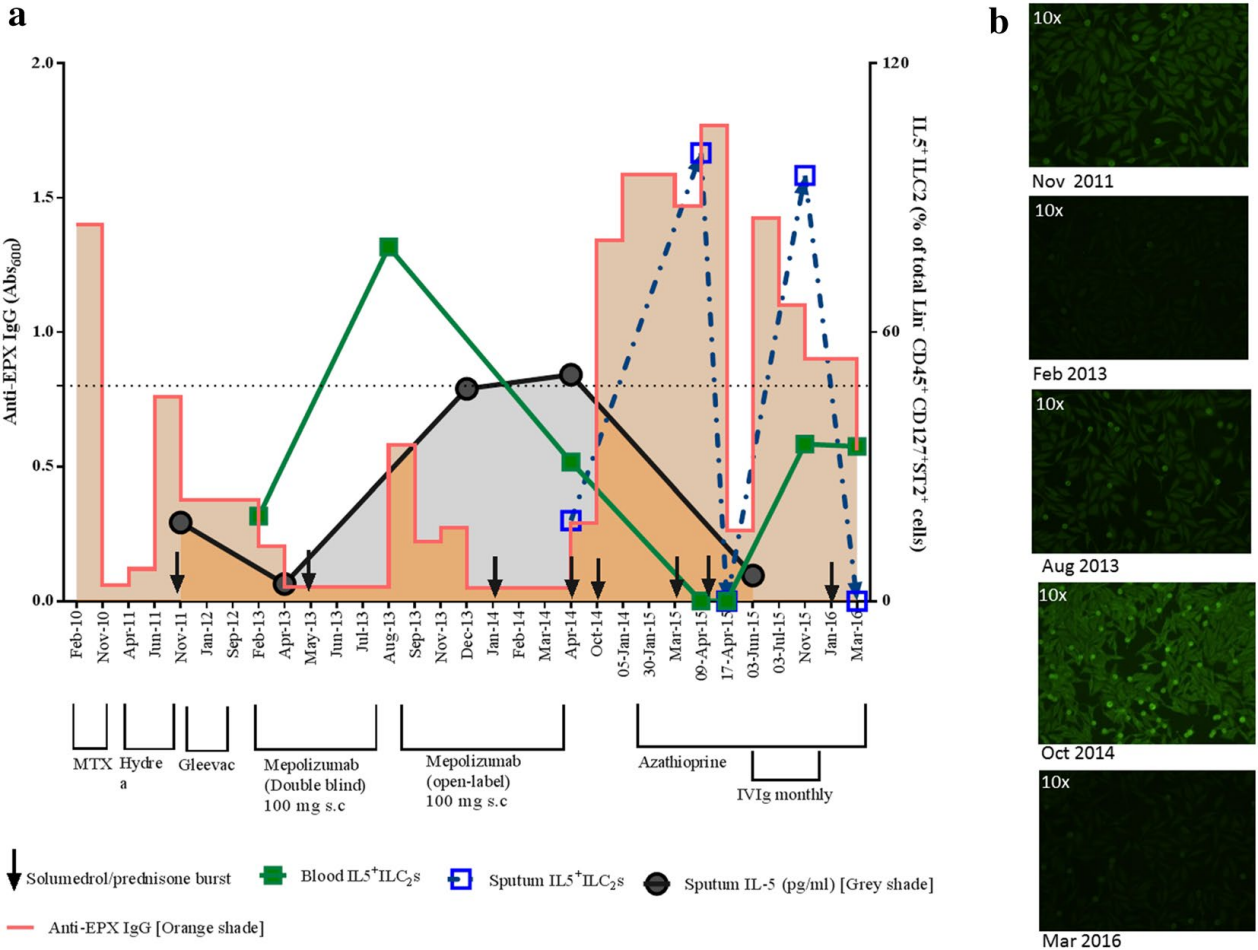
Disease progression timeline highlighting the diverse treatment and concurrent molecular events. **a** Both circulating and sputum IL5^+^ ILC2s is plotted as a percentage of a total number of enumerated ILC2s (protocol described in [5]), along with sputum IL-5 levels (see Additional file 1: online repository for methods) on the *right Y axis*. **b** Representative images of the ANA reactivities of sputum samples collected at the given dates is presented here (see Additional file 1: online repository for method and validation)

Our molecular studies demonstrated that sputum IL-5 levels peaked at the end of her Mepolizumab therapy (Fig. 2). This increase in IL-5 during an anti-IL5 therapy, along with demonstrable sputum eosinophils, can be explained by IL-5/Mepolizumab ICs acting as ‘cytokine depots.’ Early studies in murine models from the 1990s report that cytokine: anti-cytokine complexes exhibit increased in vivo half-lives, functional potency, and downstream biological activity of the bound cytokine [4]. This increase in in vivo potency/downstream biological activity by cytokine: anti-cytokine mAb complexes has been demonstrated for several cytokines viz., IL-2 [6], IL-4 [7], IL-6 [8], IL-7 [9]. Indeed, by using a sandwich ELISA (see Additional file 1: online repository for methods), we could detect IL-5 bound to immunoglobulins immunoprecipitated from the sputum supernatant induced at the Aug-2013 visit, post 6 infusions (Visit 9, #MEA115575), otherwise undetectable in prior samples (Additional file 1: Figure S2).

We enumerated IL-5^+^ ILC2 cells by flow cytometry in both blood and sputum. There was a dramatic increase in circulating IL5^+^ ILC2 s after six injections of Mepolizumab (Aug-13, Fig. 2). By Apr-14, sputum IL5^+^ ILC2 s was demonstrable despite further nine infusions of s.c 100 mg Mepolizumab (Fig. 2). A recent study investigated the cause of rare eosinophilia post-low dose IL-2 therapy in HCV-induced vasculitis. This was shown to be an IL-2/anti-IL-2 mAb (IC)-mediated mechanism where the increased bioavailability of the bound IL-2 favoured interactions with IL-2R expressed by ILC2 s, stimulating in situ IL-5 production [10]. Further, ICs formed between an allergen, and its specific IgG can be intercepted by FcγRIII expressed on antigen-presenting cells, which leads to an increase in Th2 signalling through up-regulation of IL-33 [11]. In fact, this can be extrapolated to any IC with IgG, since the up-regulation of IL-33 is mediated by the Fc:FcR binding and not the complexed antigen. IL-33 has been shown to activate resident ILC2 s (expressing the IL-33 receptor) to produce Th2 cytokines [11]. Therefore, an IC-mediated increase in Th2 signalling could lead to the observed airway eosinophilia via activation of IL-5 producing ILC2s, subsequent lung-homing and differentiation of EoPs, in situ.

Initial depletion of IL-5 by targeted therapy might have suppressed autoantigen-specific regulatory cells, thereby compromising the local tolerance, as per observations in a model of experimental autoimmune neuritis [12]. Being a retrospective case study, we were unable to enumerate regulatory cells pre- and post therapy (via flow cytometry) to gauge any effect of anti-IL-5 targeted therapy on regulatory lymphocyte population/activity. However, compared to data collated from 15 healthy volunteer sputum (mean IL-10 levels: 2.9 ± 1.3 pg/mL, upper 90% percentile 5.88 pg/mL), the study patient had low levels of IL-10 in sputum, that varied between 0.29 and 0.59 pg/mL in all the samples tested between Aug-2013 and Jun-2015. This was indicative of a ‘local’ micro-environment with compromised immune-regulation. Indeed, laboratory investigations, as early as 2010, showed that the patient had a propensity towards local autoimmune response [13]. We could detect high titres of anti-eosinophil peroxidase (EPX) IgG and anti-nuclear antibodies (ANAs, Figs. 1b, 2; see Additional file 1: online repository for methods) in the sputum, otherwise undetectable in circulation. Incidentally, there was also an increase documented for sputum B-cell activity (B-cell activating factor (BAFF) levels: pre-Mepolizumab, Feb-13—19.2 pg/mL to post-Mepolizumab, Oct-14: 228 pg/mL). We speculate that increase in activated ILC2s contributed to the local B-cell activity since the former has recently been reported to activate and promote survival of B-cells in vitro [14]. The concurrent increase in eosinophils and autoantibodies would allow spontaneous IgG-mediated eosinophil degranulation in the airways, an event that is known to be steroid-unresponsive [15]. Increased frequency of a steroid-unresponsive event could explain the increase in maintenance prednisone dose to 40 mg (Fig. 1b).

To target the lymphocytes, the patient was then treated with daily 150 mg azathioprine without any continual clinical improvement. At an all-time low FEV_1_ of 0.56 L (23% predicted), she was started on intravenous immunoglobulins (IVIgs, 2 g/kg weight, over 3 days, monthly) as an autoantibody mopping-up strategy. Post six monthly infusions (Nov-15) her sputum autoantibody titres reduced steadily, and FEV_1_ improved to 1.13 L (Fig. 2), suggesting a plausible underlying autoimmune-type anomaly. Indeed, the autoantibody levels correlated with the deteriorating lung-function (FEV_1_) over the entire timeline of the case study (r = −0.55, *P* = 0.01, Fig. 1b).

At the last IVIg infusion, the prednisone dose was reduced to 17.5 mg to assess a steroid-sparing effect. The lowered dose was unable to suppress the local inflammatory mediators that allowed ‘lung-homing’ of lymphocytes and increased IL5^+^ ILC2s, driving an in situ eosinophilopoeisis evident by the concomitant increase in sputum eosinophils (Nov-15 Fig. 2). The exacerbation (Jan-16) was treated with a prednisone burst. In the absence of the steroid-unresponsive IgG-induced degranulation post-IVIg (Nov-15), an ILC2-mediated increase in sputum eosinophilia recently shown to be steroid-responsive [16], could be curbed by a prednisone burst. Thereafter, the patient was maintained at 17.5 mg of daily prednisone (Fig. 1b). Her recent FEV_1_ in Mar-16 was recorded to be 1.54 L (Fig. 1b). She continues to be hyperresponsive (PC_20_ methacholine <0.03 mg/mL), contributing to her daily symptoms and poor asthma control, ACQ of 1.5). She is being considered for therapy with additional IVIg and/or intravenous reslizumab (an IgG_4_ anti-IL5 mAb, weight-adjusted dosing) or anti-IL4Rα mAb (Dupulimab).

## Conclusion

To our knowledge this is the first case report of an anti-IL-5 therapy leading to worsening of clinical symptoms in eosinophilic severe asthma. In summary, we provide evidence that normal blood eosinophil counts post low-dose Mepolizumab therapy does not confirm that the eosinophil-driven disease process is adequately controlled. Indeed, sputum was demonstrated to be superior to blood in monitoring response to therapy. Although a direct evidence of in vivo IC-mediated injury could not be demonstrated, a concurrent increase in sputum IL-5 (whether in a free-form or bound) and IL5^+^ ILC2 population provided indirect evidence of possible ICs acting as ‘cytokine depots’, supporting in situ eosinophilopoiesis. And finally, we demonstrated that Mepolizumab, at the current prescribed dose and delivery platform, may precipitate steroid-insensitivity by triggering steroid-unresponsive auto-inflammatory mechanisms, especially in patients who are predisposed to it. Further studies are necessary to understand the airway pharmacokinetics of novel mAbs, effective dosing strategies particularly whether they have to be guided by antigen-concentrations or body weight, and the appropriate therapy for patients with inadequate or worsening clinical responses with the monoclonal antibodies.

## Additional file

**Additional file 1.** Figures and immunological methods.

## Abbreviations

mAb: monoclonal antibody
IC: immune complex
FEV_1_: forced expiratory volume in 1 s
s.c.: subcutaneous
EPX: eosinophil peroxidase
IP-Igs: immunoprecipitated immunoglobulin
IVIg: intravenous immunoglobulin
ANA: anti-nuclear antibody
BAFF: B-cell-activating factor of the TNF family
BAL: broncho-alveolar lavage
IL: interleukin
Ig: immunoglobulin
ILC2: innate lymphoid cells of group 2
