# Supplementary material for: Airway autoimmune responses in severe eosinophilic asthma following low-dose Mepolizumab therapy
Source: Allergy Asthma Clin Immunol. 2017 Jan 6;13:2. doi: 10.1186/s13223-016-0174-5 (PMC5216532; doi:10.1186/s13223-016-0174-5)
Supplement: Supplementary file 1 — Additional file 1. Figures and immunological methods. [file 13223_2016_174_MOESM1_ESM.docx]

**Online Figure Legend:**

**Figure S1: Physiological consequence of *in situ* IC formation due to low dose mAb therapy:** An adapted model based on the classical antibody-antigen precipitation curve based on the Precipitin test first demonstrated by Kraus Z, Matĕja F,1966, *Acta Derm Venereol.* 1966; 46(2):217-223, highlighting the ‘zone of equivalence’ in mAb therapy.


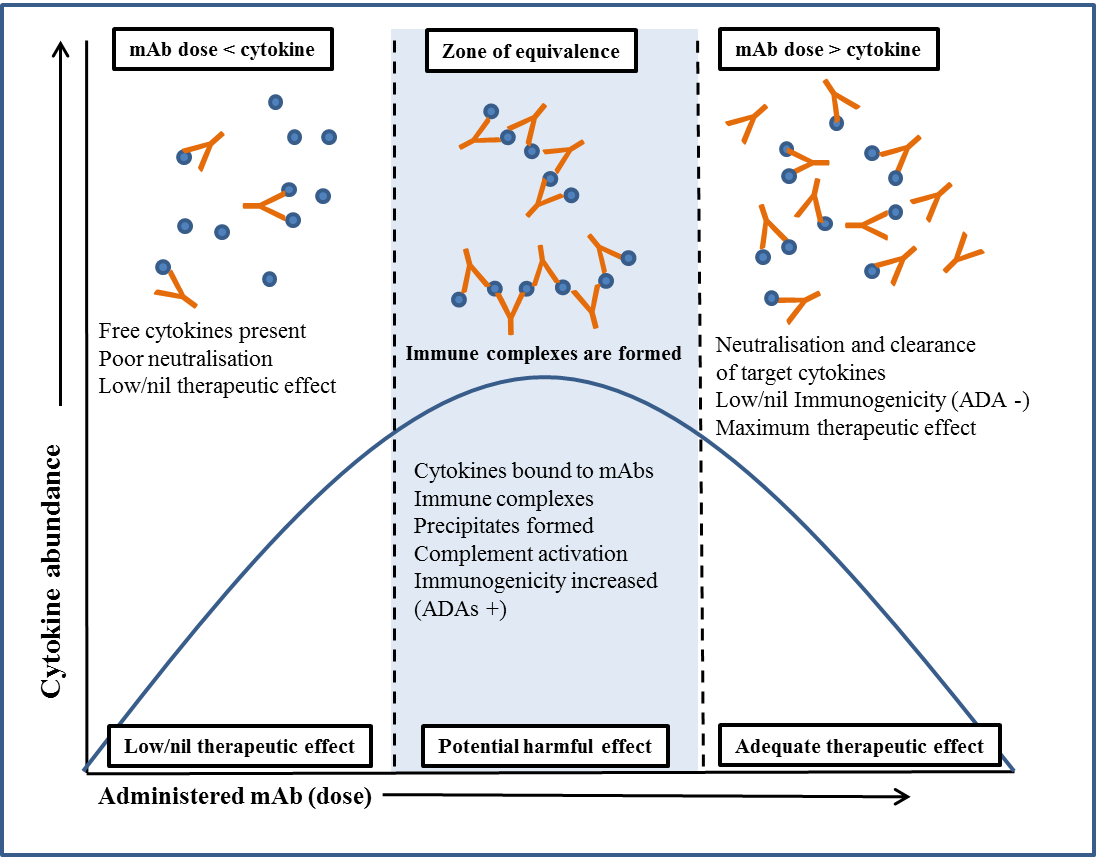


**Figure S2: Detectable IL-5 in immunoprecipitated immunoglobulins from sputum:**  IL-5 (pg/ml) levels detectable in immunoprecipitated-immunoglobulins (IP-Igs) eluted from sputum supernatants, plotted for the time points where adequate volume was available for measurements. N.B. Feb-2013 marked the start of the Mepolizumab 100 mg subcutaneous double blind study (Visit 1), while Dec-2013 marks last visit (Visit 9) post 6 infusions of s.c. 100 mg Mepolizumab.

**
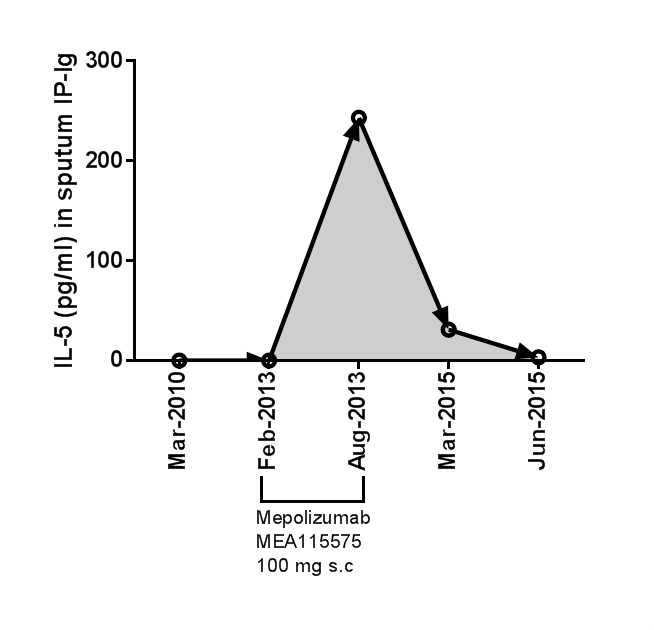
**

**Online Methods:**

***Immunoprecipitation***

To achieve an optimum signal-to-noise ratio and counteract interferences from unknown soluble fractions (and endogenous peroxidases themselves), the total immunoglobulin from sputum supernatants (the cell-free soluble fraction of the processed sputum) were immunoprecipitated using Pierce™ Protein A/G Agarose (Thermo Fisher Scientific, MA, USA). 100 µL of sputum supernatant was optimised for 30 µL of resin slurry suspended in 100 µL binding buffer (0.1 M Sodium phosphate, 0.15 NaCl, pH 7.4). The 200 µL suspension was incubated on a rotator at 4˚C for an hour to allow thorough mixing of the protein beads and the sputum Igs. The beads were washed thrice with a high salt buffer (500 nM KCl, 0.1% Triton-X100, 20 mM Tris, pH 7.4) and eluted with 0.1 M Glycine, HCl (pH 2.60). An equal volume of 1M Tris (pH7.4) was added to neutralize. A 2 fold dilution was maintained for all samples by keeping the elution volume constant at 200 µl.

***ELISA for detection of Anti-EPX antibodies***

For assessing the anti-EPX reactivity and nature of the detected antibody, the Maxisorb plates were coated with 1 µg/mL of recombinant EPX (LEE Biosolutions, MO, USA) for 1.5 hrs at 4˚C. The blocked plates were incubated overnight at 4˚C with 40 µL of freshly eluted IP at a dilution of 1:2 in separate wells in duplicate. Biotinylated secondary antibodies (BD Biosciences, ON, Canada) against human IgG (1:2000 dilution), IgM (1:1000) and IgA (1:1000) were added to the respective wells for 1 hr at RT, and subsequently, the plates were developed and absorbance read at 600 nm. Considering the eight times weight/volume dilution of the selected sputum during processing, and two-fold dilution during IP, the final dilution at which all samples were tested was 1:32. The cut-off threshold (marked as dotted lines on Figure 1B, and 2) is calculated based on the average readings of 15 healthy volunteers ± 2 times the standard deviation.

***Anti-Nuclear Antibody testing in sputum***

The presence of ANAs from the sputum supernatants was evaluated using a clinical diagnostic kit (Immco Diagnostics, Buffalo, New York, USA) and further validated by a Line Immunoassay kit (IMTEC-ANA-LIA-Maxx, ITC 92005, Human Worldwide, Germany). The Immunofluorescence reactivity of sputum supernatant against Hep-2 cells were examined with a Nikon Eclipse TE2000-E (Melville, New York, USA) microscope, images acquired with Q-imaging Retiga 2000R camera (Surrey, British Columbia, Canada) and analysed by NIS-Elements software (version 3.0AR, Melville, NY, USA).

***Sputum Cytokine detection***

Sputum IL-5 was measured by IL-5 duo-set ELISA, R&D Systems, MN, USA; developed by BluePhos substrate kit, KPL Inc., MA, USA. BAFF concentration in the sputum supernatants were quantified using commercially available Quantikine ELISA kit (R&D Systems, Minnesota, USA). 25µl of supernatant were diluted with 25µl of the assay calibrator, and the rest of the protocol was followed as instructed. The kit was validated to negate out the interference of endogenous EPX with the HRP-based detection system by spiking the standards with 1 ng of EPX. The observed values were within 2 standard deviation of the original values.

***Immunecomplexed-IL-5 detection***

To estimate if there were any IL-5 complexed with immunoglobulins as a measure of plausible ‘immune-complexes’ *in situ*, we used the immunoprecipitated sputum immunoglobulins as ‘sample’ in an IL-5 sandwich ELISA (R&D systems, Minnesota, USA). Post-incubation with the biotinylated detection antibody, the protocol was adapted for an alkaline phosphatase based detection system (BluePhos® Microwell substrate, KPL Inc., MA, USA) to eradicate any interference from complexed EPX in the immunoprecipitated eluate used as sample. Results were plotted as pg/ml, extrapolated from the standard curve.

Graphpad Prism version 7.00 (La Jolla, CA, USA) has been used for all statistical analysis.
